# Enhanced Numerical Modeling of Non-Newtonian Particle-Laden Flows: Insights from the Carreau–Yasuda Model in Circular Tubes

**DOI:** 10.3390/polym18010043

**Published:** 2025-12-23

**Authors:** Medeu Amangeldi, Dongming Wei, Asma Perveen, Dichuan Zhang

**Affiliations:** 1 Department of Mathematics, School of Sciences and Humanities, Nazarbayev University, Astana 010000, Kazakhstan; medeu.amangeldi@nu.edu.kz; 2 Department of Mechanical & Aerospace Engineering, School of Engineering and Digital Sciences, Nazarbayev University, Astana 010000, Kazakhstan; asma.perveen@nu.edu.kz; 3 Department of Civil & Environmental Engineering, School of Engineering and Digital Sciences, Nazarbayev University, Astana 010000, Kazakhstan; dichuan.zhang@nu.edu.kz

**Keywords:** particle-laden flows, rheology, numerical modeling, Carreau–Yasuda model, suspension dynamics

## Abstract

Particle-laden flows in non-Newtonian fluids are encountered in a variety of industrial applications, such as concrete pumping and battery electrode slurry processing, where accurate prediction of particle migration is essential for performance and product quality. This work investigates fully developed suspension flows in circular tubes, combining the shear-induced diffusion framework of Phillips et al. with the Krieger–Dougherty relative viscosity and the Carreau–Yasuda constitutive model. Unlike previous studies that generally rely on Newtonian or simple non-Newtonian rheology models, we employ the Carreau–Yasuda model, a more sophisticated constitutive relation that captures both shear-thinning behavior and Newtonian plateau regimes. By applying nondimensionalization and variable transformations, we reduce the governing coupled differential equations to a system of nonlinear algebraic equations, which allows for efficient computation of both particle concentration and velocity profiles. A systematic parametric study was conducted to evaluate the influence of several factors, including the pressure gradient, average particle concentration, and the five parameters of the Carreau–Yasuda model. Additionally, the migration parameter $\alpha =K_{c}/K_{\eta }$ was considered. The results reveal how the increased rheological complexity of the Carreau–Yasuda model significantly alters the migration dynamics when compared to simpler models. These novel findings have direct implications for optimizing industrial processes involving highly loaded suspensions, offering more accurate predictions of particle behavior under varying flow conditions. For the validation of our findings, experimental data in the literature was used.

## 1. Introduction

Fluid flows with suspended particles are encountered in many industrial applications, such as concrete pumping [1] and extrusion and injection molding processes with filled polymers [2,3]. Understanding the fundamentals of these flows through numerical modeling is therefore essential. In particular, it is important to investigate the behavior of suspension flows using generalized rheological models, which offer a broader range of applicability.

In this work, we focus on steady-state flows in circular tubes with non-Newtonian fluids. We present a simplified approach to numerically modeling the particle volume fraction and velocity distributions based on a reduced form of the shear-induced diffusion equation. We then examine the influence of both physical parameters and the rheological parameters of the Carreau–Yasuda model on concentration and velocity profiles.

One important application of such studies is the rheology of electrode slurries, which has a critical impact on the final coating microstructure. Challenges include high slurry viscosity, which increases pumping pressure and limits coating speed; elasticity, which can lead to instabilities and defects; and excessive flow, which may result in slumping and poorly structured coatings. These complexities highlight the importance of optimizing slurry formulations. However, the wide range of solvent systems and components involved makes it difficult to identify the exact origins of these unfavorable rheological properties [4]. Furthermore, precise measurements of extensional properties using miniature rheometer have revealed significant variability in slurry behavior across different formulations and binder systems [5].

The work of Phillips et al. [6] provides a fundamental reference for this study. They derived the shear-induced diffusion equation for the particle volume fraction (ϕ) and established the corresponding no-flux boundary condition. A key difference between our work and theirs is that Phillips et al. assumed Newtonian fluids in Couette and Poiseuille flows, whereas we consider non-Newtonian flows governed by the Carreau–Yasuda model [7].

Following the seminal work of Phillips et al. [6], a substantial body of literature has focused on the theoretical and numerical modeling of shear-induced particle migration without direct experimental validation, largely due to the limited availability of comprehensive experimental data for complex suspension flows. Such pure modeling studies have nonetheless played a central role in advancing the understanding of suspension dynamics. For instance, Kang and Mirbod [8] employed direct numerical simulations to investigate shear-induced migration in Poiseuille and circular Couette flows of semi-dilute and concentrated Brownian suspensions with shear-thinning behavior. Dbouk et al. [9] studied particle migration using the Suspension Balance Model and finite-volume simulations, examining the influence of particle stress formulations on migration predictions. More recently, Kauzlarić et al. [10] proposed a non-local extension of the Phillips model, demonstrating how alternative modeling assumptions affect concentration profiles. These works illustrate that modeling-based investigations, even in the absence of new experimental data, constitute a well-established and widely accepted approach in the study of shear-induced particle migration.

Earlier, Chen et al. [11] investigated shear-induced particle migration in non-Newtonian flows of nickel-powder-filled polymers. They employed the power-law model to describe the viscosity–shear rate relation and incorporated temperature dependence using the Arrhenius law. Solving the coupled differential equations for velocity and particle volume fraction proved to be nontrivial. Subsequently, Chen et al. [12] developed a numerical scheme to obtain these solutions for both steady and unsteady cases. Their method combined the Newton–Raphson iterative approach for velocity fields with a finite-difference semi-implicit scheme (Crank–Nicolson [13]) for the diffusion equation.

In contrast, we applied nondimensionalization and variable transformations, following techniques presented by Wang [14], to reduce the governing system of differential equations to a system of nonlinear algebraic equations. The solutions were then obtained numerically, which is computationally less intensive than differential equation–based approaches. Moreover, the Carreau–Yasuda model provides a more general description compared to the power-law model.

The originality of the present work lies in (i) the incorporation of the Carreau–Yasuda constitutive model within the shear-induced diffusion framework, enabling the treatment of shear-thinning fluids with finite viscosity plateaus; (ii) the reduction of the governing equations to a system of nonlinear algebraic equations through nondimensionalization and variable transformation, resulting in a computationally efficient solution strategy (see Appendix A); and (iii) a systematic parametric investigation encompassing physical parameters, particle migration coefficients, and all rheological parameters of the Carreau–Yasuda model (see Results and Discussion section and Appendix B).

Several related studies have examined particle migration in different flow configurations. Choi et al. [15] studied concrete pumping in pipes using the Bingham fluid model for the viscosity–shear rate relation and commercial CFD software (Fluent) for numerical solutions. Siqueira and Carvalho [16] investigated particle migration in planar extrudate flows of suspensions of hard spheres. They analyzed Newtonian flows between parallel plates and subsequent free-surface extrudate flows, employing a stabilized finite element method along with elliptic mesh generation to compute the free surface. Rebouças et al. [17] studied fully developed suspension flows in circular tubes, focusing on the effects of particle and shear-induced viscosity. Their work is closely related to ours, as they also solved the shear-induced diffusion equation for non-Newtonian flows. They examined the effects of the average particle volume fraction on concentration and velocity profiles, and their results agree qualitatively with our findings. However, their approach addressed the singularity near the tube centerline and relied on the Cross model for fluid viscosity, whereas we adopt the more general Carreau–Yasuda model, which includes an additional parameter (*a*). Furthermore, we systematically explore the influence of a broader set of parameters, including the pressure gradient, average particle volume fraction, all five Carreau–Yasuda parameters, and the model parameter $K_{c}/K_{\eta }$ (hereafter α).

In addition to numerical studies, Rueda [18] presented a comprehensive experimental investigation of the rheological properties of suspension flows, examining how particle size, concentration, and fiber interactions influence viscosity and flow regimes. His results highlight the sensitivity of suspension rheology to microstructural features, providing valuable experimental context for the numerical analysis carried out in the present work. In particular, these findings reinforce the importance of understanding how rheological parameters and particle migration mechanisms shape the concentration and velocity profiles studied here.

It is also important to emphasize that our approach has been verified against existing models in the literature. Specifically, back-substitution of the computed particle volume fraction and shear-rate distributions into the governing equations yields negligible residual errors, confirming the correctness of the formulation and implementation. Moreover, qualitative agreement with the results of Rebouças et al. [17] further supports the validity of our model. To further validate our numerical framework, we compared our predicted velocity profiles against experimental data from Preziosi et al. [19] for low-viscosity emulsions flowing in a confined capillary. The comparison shows good agreement, with a mean relative error of less than 8%, thereby demonstrating the applicability of our Carreau–Yasuda-based model to real experimental systems.

The remainder of this paper is organized as follows. Section 2.1, Section 2.2 and Section 2.3 present the mathematical formulation of the problem, including the shear-induced diffusion model and the Carreau–Yasuda constitutive relation. Section 2.4 describes the nondimensionalization, numerical scheme, and iterative algorithm used to obtain the solutions. Section 3 discusses the numerical results, highlighting the effects of physical and rheological parameters on particle concentration and velocity profiles. Finally, Section 4 summarizes the main findings and outlines the implications of this modeling framework for future studies and applications.

## 2. Model and Methods

### 2.1. Governing Equations

The flow is governed by equation of continuity for an incompressible fluid

(1) ∇·v=0

the conservation equation of linear momentum

(2) ∇·τ+∇p=0

and Newton’s Law of viscosity

(3) $$\tau =\eta \left(\dot{\gamma }\right)\dot{\gamma }$$

### 2.2. Particle Poiseuille Flow Through a Cylindrical Tube

**Figure 1:**
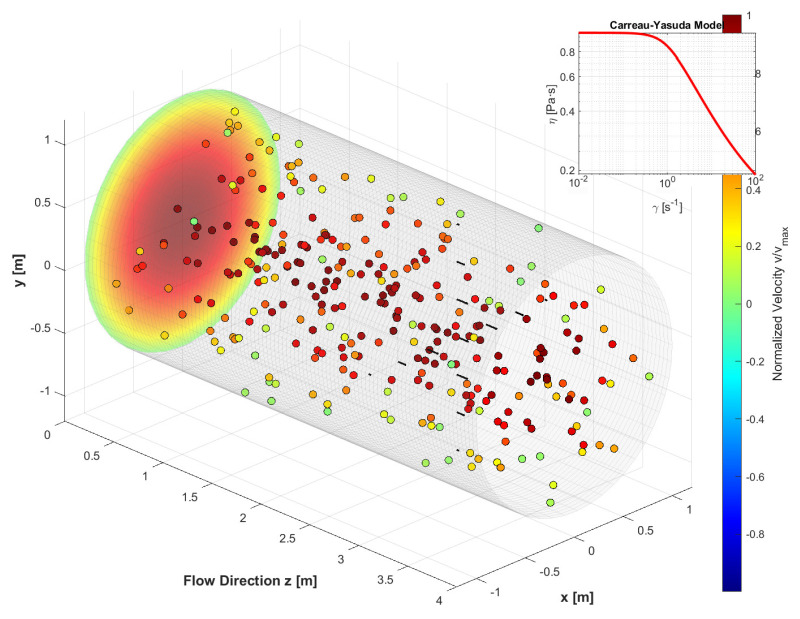
Schematic representation of steady pressure-driven flow of a particle-laden non-Newtonian suspension in a circular tube.

We consider the flow of a particle-laden non-Newtonian suspension in a circular tube under steady pressure-driven conditions (Poiseuille flow). The central modeling challenge is to describe how particles migrate across streamlines due to hydrodynamic interactions and spatial variations in viscosity. To this end, we adopt a shear-induced diffusion framework originally proposed by Phillips et al. [6], which balances the competing effects of particle collisions, viscosity gradients, and Brownian motion. Figure 1. is used as a schematic illustration of the anticipated results of our numerical simulation.

Let ϕ(r,t) denote the particle volume fraction. To describe the rheology of the suspension, we combine a concentration-dependent relative viscosity model with a shear-rate-dependent constitutive law for the carrier fluid. Consider the Krieger & Dougherty [20] model, which is typically applied for the particle size range between nano- and micrometers, for laminar flow with the following terms on particle-volume-induced relative viscosity:

(4) $$\begin{array}{c} \begin{array}{cccc} \eta (\phi ,\dot{\gamma }) & = & \eta_{r}\left(\phi \right)\;\eta_{s}\left(\dot{\gamma }\right) & \mathrm{viscosity}\mathrm{of}\mathrm{concentrated}\mathrm{suspension} \end{array} \end{array}$$

(5) $$\begin{array}{c} \begin{array}{cccc} \eta_{r}\left(\phi \right) & = & {\left(1-\phi /\phi_{m}\right)}^{-1.82} & \mathrm{relative}\mathrm{viscosity} \end{array} \end{array}$$

(6) $$\begin{array}{c} \begin{array}{cccc} \eta_{s}\left(\dot{\gamma }\right) & = & \eta_{\infty }+\left(\eta_{0}-\eta_{\infty }\right){\left(1+{\left(\lambda \dot{\gamma }\right)}^{a}\right)}^{\frac{n-1}{a}} & \mathrm{viscosity}\mathrm{of}\mathrm{media} \end{array} \end{array}$$

(7) $$\begin{array}{c} \begin{array}{cccc} N_{c} & = & -K_{c}b^{2}\phi \nabla \left(\dot{\gamma }\phi \right) & \mathrm{flux}\mathrm{by}\mathrm{varying}\mathrm{collision} \end{array} \end{array}$$

(8) $$\begin{array}{c} \begin{array}{cccc} N_{\eta } & = & -K_{\eta }b^{2}\dot{\gamma }\phi^{2}\frac{\nabla \eta }{\eta } & \mathrm{flux}\mathrm{by}\mathrm{spatially}\mathrm{varying}\mathrm{viscosity} \end{array} \end{array}$$

(9) $$\begin{array}{c} \begin{array}{cccc} N_{b} & = & -D\nabla \phi & \mathrm{Brownian}\mathrm{diffusion}\mathrm{of}\mathrm{particles} \end{array} \end{array}$$

Fluxes $N_{c}$ and $N_{\eta }$ represent shear-induced migration mechanisms introduced by Phillips et al. [6], while $N_{b}$ accounts for random particle motion due to Brownian effects. The symbol *b* in the particle flux terms $N_{c}$ and $N_{\eta }$ denotes the particle radius (m). Unless otherwise indicated, $K_{c}$ and $K_{\eta }$ are taken as dimensionless migration coefficients (Phillips et al. formulation) so that the combination $K_{c}b^{2}$ and $K_{\eta }b^{2}$ has dimensions of $m^{2}$ (as needed for a diffusive flux).

The Brownian diffusion coefficient *D* has units of $m^{2}/s$, and the Peclet number is defined as

$$\mathrm{Pe}\equiv \frac{\dot{\gamma }b^{2}}{D}.$$

This number Pe measures the relative strength of shear-induced transport compared to Brownian motion. Thus, the conservation equation for the particle volume fraction becomes

(10) $$\begin{array}{c} \frac{\partial \phi }{\partial t}=-\nabla \cdot \left(N_{c}+N_{\eta }+N_{b}\right). \end{array}$$

In this work we adopt the steady-state assumption Pe→∞, meaning that the Brownian flux $N_{b}$ is negligible compared to the shear-induced fluxes $N_{c}$ and $N_{\eta }$. For clarity, we explicitly state the range of Peclet numbers for which this approximation is valid. The Peclet number quantifies the competition between shear-driven migration and Brownian diffusion. For colloidal particles, Brownian contributions are significant when Pe≲1 and become negligible only when $\mathrm{Pe}≳10^{2}$–$10^{3}$, as reported in Phillips et al. [6] and subsequent experimental studies on shear-induced migration. For the particle sizes and shear rates relevant to the systems modeled here (typically b=1–10μm and $\dot{\gamma }=10^{2}$–$10^{3}\;s^{-1}$), the resulting Peclet numbers fall in the range $\mathrm{Pe}∼10^{3}$–$10^{7}$. Thus, Brownian diffusion is several orders of magnitude weaker than the hydrodynamic migration mechanisms, and neglecting $N_{b}$ is fully justified for the parameter regime considered in this study. Thus, replacing the flux terms in (10) with Equations (4)–(9) gives us

(11) $$\begin{array}{c} \frac{\partial \phi }{\partial t}-b^{2}\nabla \left[K_{c}\phi \nabla \left(\dot{\gamma }\phi \right)+K_{\eta }\dot{\gamma }\phi^{2}\frac{\nabla \eta }{\eta }\right]=0 \end{array}$$

For flows in a cylindrical tube, the gradient can be written in $\nabla =\left(\frac{\partial }{\partial r}+\frac{\partial }{\partial z}\right)$ in general form. At the tube wall, particle conservation requires that the net flux is normal to the boundary vanishes, leading to the no-flux condition

(12) $$\begin{array}{c} n\cdot \left[K_{c}\phi \nabla \left(\dot{\gamma }\phi \right)+K_{\eta }\dot{\gamma }\phi^{2}\frac{\nabla \eta }{\eta }\right]=0 \end{array}$$

So, combining (11), (12), and the governing Equations (1)–(3), we get the generalized system of differential equations of velocity profile and particle volume fraction distribution, which is

(13) $$\begin{array}{ccc} \frac{\partial p}{\partial z}+\frac{1}{r}\frac{\partial }{\partial r}\left(r\dot{\gamma }\eta \right) & = & 0\\ \frac{\partial \phi }{\partial t}-b^{2}\nabla \left[K_{c}\phi \nabla \left(\dot{\gamma }\phi \right)+K_{\eta }\dot{\gamma }\phi^{2}\frac{\nabla \eta }{\eta }\right] & = & 0 \end{array}$$

with the no-flux boundary condition at the wall

(14) $$\begin{array}{c} n\cdot \left[K_{c}\phi \nabla \left(\dot{\gamma }\phi \right)+K_{\eta }\dot{\gamma }\phi^{2}\frac{\nabla \eta }{\eta }\right]=0 \end{array}$$

We note that the phrase “Newton’s law of viscosity” in the text is used to refer to the kinematic relation between shear stress and shear rate. Strictly speaking, Newton’s law requires a constant viscosity (Newtonian fluid), $\tau =\eta \;\dot{\gamma }$ with η= const. In this work we instead adopt the generalized Newtonian form

$$\tau =\eta \left(\dot{\gamma },\phi \right)\;\dot{\gamma },$$

where the scalar viscosity η depends on the local shear rate and particle concentration through the constitutive relations (Krieger–Dougherty for the concentration dependence and Carreau–Yasuda for the shear-rate dependence). Thus there is no inconsistency: the momentum equation, which is the first equation in (13), is written for the generalized-Newtonian stress $\eta \left(\dot{\gamma },\phi \right)\dot{\gamma }$.

Regarding the pressure gradient appearing first equation in (13), in the fully developed pipe flows considered here, the axial pressure gradient ∂p/∂z is spatially uniform and acts as the external driving force. In our computations it is treated as the prescribed driving parameter (related to the imposed pressure drop $P_{0}-P_{L}$ over the pipe length *L*), but equivalently one can prescribe a total flow rate and determine the corresponding ∂p/∂z. The wall force balance then yields

$$\eta_{w}{\dot{\gamma }}_{w}=-\frac{dp}{dz}\frac{R}{2},$$

which connects the (dimensional) pressure gradient to the wall shear rate used for nondimensionalization.

### 2.3. Steady State Velocity and Particle Distributions

In steady state flow, $\frac{\partial \phi }{\partial t}=0$, and we assume the *r*-direction, so the second equation in the coupled system (13) becomes

(15) $$\begin{array}{c} \frac{\partial }{\partial r}\left[K_{c}\phi \frac{\partial }{\partial r}\left(\dot{\gamma }\phi \right)+K_{\eta }\dot{\gamma }\phi^{2}\frac{1}{\eta }\frac{\partial \eta }{\partial r}\right]=0 \end{array}$$

which can be integrated over *r*, and applying the no-flux boundary condition to compute the constant of integration, we get

(16) $$\begin{array}{c} K_{c}\phi \frac{\partial }{\partial r}\left(\dot{\gamma }\phi \right)+K_{\eta }\dot{\gamma }\phi^{2}\frac{1}{\eta }\frac{\partial \eta }{\partial r}=0 \end{array}$$

which means no flux everywhere in the suspension in the steady-state scenario. This equation can be integrated with the boundary condition at the wall (subindex *w*), so we get the following simplification

(17) $$\begin{array}{c} \frac{\dot{\gamma }\phi }{{\dot{\gamma }}_{w}\phi_{w}}-{\left(\frac{\eta_{w}}{\eta }\right)}^{K_{\eta }/K_{c}}=0 \end{array}$$

Now we want to simplify the system. For steady, axisymmetric, fully developed flow in a cylindrical tube (with $u_{r}=0$, $u_{\theta }=0$), the radial momentum balance reduces to ∂p/∂r=0. Hence the pressure depends only on the axial coordinate, p=p(z). By rearrangement of the first part of the coupled system (13), we get

(18) $$\begin{array}{c} \frac{\partial }{\partial r}\left(r\dot{\gamma }\eta \right)=-\frac{\partial p}{\partial z}r \end{array}$$

and integration of (18) gives us

(19) $$\begin{array}{c} \dot{\gamma }\eta =-\frac{\partial p}{\partial z}\frac{r}{2}+C \end{array}$$

Applying the regularity condition at r=0 gives C=0. Then, we have the simplified equation

(20) $$\begin{array}{c} \dot{\gamma }\eta =-\frac{\partial p}{\partial z}\frac{r}{2} \end{array}$$

Summarizing the coupled ODE for steady-state case, we have the following system of differential equations (see details in Appendix C)

(21) $$\begin{array}{ccc} \dot{\gamma }\eta +\frac{\partial p}{\partial z}\frac{r}{2} & = & 0\\ \frac{\dot{\gamma }\phi }{{\dot{\gamma }}_{w}\phi_{w}}-{\left(\frac{\eta_{w}}{\eta }\right)}^{K_{\eta }/K_{c}} & = & 0 \end{array}$$

### 2.4. Nondimensionalization and Numerical Details

In order to further simplify the coupled system (21), we introduce the nondimensional variables as follows

(22) $$\begin{array}{c} \begin{array}{cccc} \tilde{r} & = & r/R & \in [0,1]\\ s & = & \dot{\gamma }/{\dot{\gamma }}_{w} & \in [0,1]\\ \tilde{\eta } & = & \eta /\eta_{w} & \in [1,+\infty ] \end{array} \end{array}$$

Substituting the new variables (22) into (21) gives us the system in nondimensional form as

(23) $$\begin{array}{ccc} \eta_{w}{\dot{\gamma }}_{w}s\tilde{\eta }+\frac{dp}{dz}\frac{\tilde{r}R}{2} & = & 0\\ s\frac{\phi }{\phi_{w}}-{\left(\frac{1}{\tilde{\eta }}\right)}^{K_{\eta }/K_{c}} & = & 0 \end{array}$$

Note that, in our formulation, the quantity $\frac{dp}{dz}$ appears only as a constant driving term, and the equation of (23) is nondimensional relative to the other variables because the combination $\frac{dp}{dz}\;\frac{R}{2}$ naturally scales with the characteristic shear stress $\eta_{w}{\dot{\gamma }}_{w}$. Since we have s=1, $\tilde{\eta }=1$, and $\tilde{r}=1$ at the wall, the first equation in (23) becomes

(24) $$\eta_{w}{\dot{\gamma }}_{w}=-\frac{dp}{dz}\frac{R}{2}=\left(\frac{P_{0}-P_{L}}{2L}\right)R$$

where $\frac{dp}{dz}=\left(P_{0}-P_{L}\right)/L>0$ is the magnitude of the pressure gradient. Suppose we use the Krieger–Dougherty equation for $\eta_{r}\left(\phi \right)$ and the Carreau–Yasuda model for $\eta_{s}\left(\dot{\gamma }\right)$ as in Equation (6), we have

(25) $$\eta_{w}=\eta_{w}\left({\dot{\gamma }}_{w},\phi_{w}\right)=\eta_{r}\left(\phi_{w}\right)\eta_{s}\left({\dot{\gamma }}_{w}\right)$$

Combining Equations (24) and (25) gives

(26) $$\eta_{r}\left(\phi_{w}\right)\eta_{s}\left({\dot{\gamma }}_{w}\right){\dot{\gamma }}_{w}=\left(\frac{P_{0}-P_{L}}{2L}\right)R$$

The value $\phi_{m}=0.68$ is used in the Krieger–Dougherty relation because it represents the maximum volume fraction attainable in random close packing (RCP) of spherical particles. This is the limit beyond which the viscosity diverges due to geometric crowding, preventing further flow. This value is physically realistic for disordered suspensions under shear and aligns with experimental observations of dense colloidal and granular flows. Using $\phi_{m}=0.68$ ensures that the model remains consistent with the assumption of a non-crystalline, randomly packed suspension. Given $\phi_{w}$, $(P_{0}-P_{L})/L$, and *R*, we can obtain ${\dot{\gamma }}_{w}$ by solving the above nonlinear Equation (Equation (26)) for ${\dot{\gamma }}_{w}$. Once ${\dot{\gamma }}_{w}$ is obtained, then it is straightforward to obtain $\eta_{w}$, which is used as the unit for viscosity. Using the identity shown in Equation (24), we can rewrite Equation (23) as

(27) $$\tilde{r}=s\tilde{\eta }\left(s,\phi \right)$$

(28) $$\tilde{\eta }={\left(s\phi /\phi_{w}\right)}^{-\alpha }$$

where $\alpha =K_{c}/K_{\eta }$ and is taken to be 0.66 in this work, following Phillips et al. [6]. Again, using the Krieger–Dougherty equation for $\eta_{r}\left(\phi \right)$ and the Carreau–Yasuda model for $\eta_{s}\left(\dot{\gamma }\right)$, we have

(29) $$\tilde{\eta }=\eta /\eta_{w}=\eta_{r}\left(\phi \right)\eta_{s}\left(\dot{\gamma }\right)/\eta_{w}=\eta_{r}\left(\phi \right){\tilde{\eta }}_{s}\left(s\right)$$

where

(30) $${\tilde{\eta }}_{s}\left(s\right)={\tilde{\eta }}_{\infty }+\left({\tilde{\eta }}_{0}-{\tilde{\eta }}_{\infty }\right){\left[1+{\left(\tilde{\lambda }s\right)}^{a}\right]}^{(n-1)/a}$$

Here, ${\tilde{\eta }}_{0}=\eta_{0}/\eta_{w}$, ${\tilde{\eta }}_{\infty }=\eta_{\infty }/\eta_{w}$, and $\tilde{\lambda }=\lambda {\dot{\gamma }}_{w}$. The $\tilde{\lambda }$ term is the time constant λ in dimensionless form, using ${\dot{\gamma }}_{w}^{-1}$ as the characteristic time scale. Combining Equations (27) and (28), we have

(31) $$f_{1}\left(\tilde{r},s,\phi \right)=\tilde{r}-s^{1-\alpha }{\left(\phi /\phi_{w}\right)}^{-\alpha }$$

and combining Equations (27)–(29), we have

(32) $$f_{2}\left(\tilde{r},s,\phi \right)=\tilde{r}-s\eta_{r}\left(\phi \right){\tilde{\eta }}_{s}\left(s\right)$$

From the above two equations, Equations (31) and (32), one can easily obtain the volume fraction $\phi (\tilde{r})$, the shear rate distribution $s(\tilde{r})$, and the volume fraction as a function of the shear rate, ϕ(s). The velocity distribution can be obtained using the method of Wang [14] as follows. Using $R{\dot{\gamma }}_{w}$ as the unit for velocity, i.e., $\tilde{u}=v/\left(R{\dot{\gamma }}_{w}\right)$, we have

(33) $$\frac{d\tilde{u}}{d\tilde{r}}=-s$$

Since the method of Wang [14] suggests $\tilde{s}\left(\tilde{r}\right)\in \left[0,1\right]$, the velocity profile $\tilde{u}\left(\tilde{r}\right)$ is obtained by integrating the corresponding ordinary differential equation numerically in the radial direction. In this work, we employ the classical fourth-order Runge–Kutta method with a uniform radial grid and the boundary condition $\tilde{u}\left(0\right)=0$ at the tube centerline. This approach provides stable and accurate integration for smooth profiles and is widely used for ODEs of this type.

**Figure 2:**
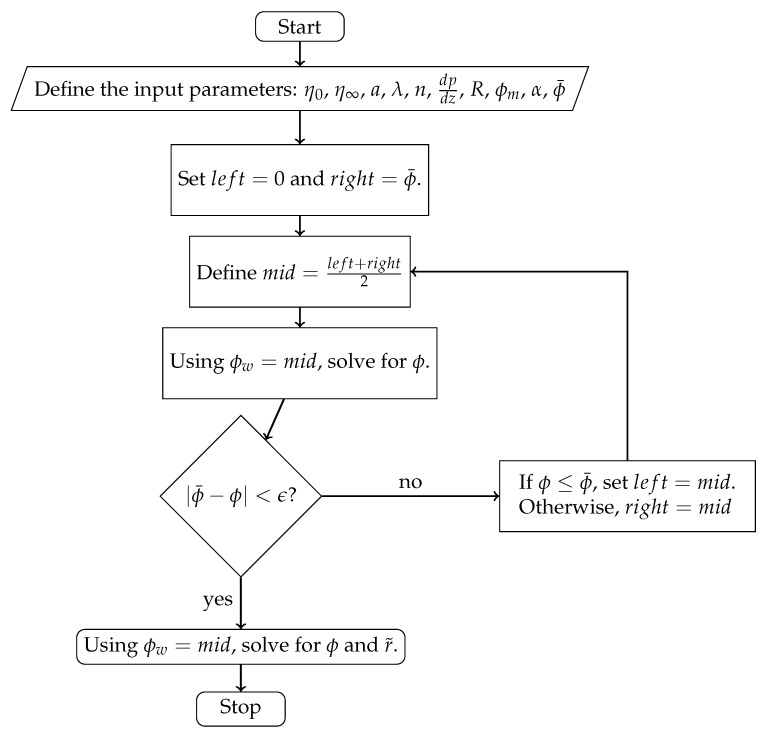
The algorithm flowchart. For each point *s*∈[0,1], we solve for ϕ(s).

If we try to solve the system, consisting of Equations (31) and (32), for *s*, complications in the numerical computations arise, for instance, ${\tilde{\eta }}_{s}\left(s\right)$ is highly nonlinear. Thus, following the idea from the paper of Wang [14], it is wise to solve for $\tilde{r}$ instead of *s*. This means, for any *s*∈[0,1], we want to solve for $\tilde{r}$ and ϕ, which can be easily obtained by root finding (ϕ) in the following equation

(34) $$s^{1-\alpha }{\left(\phi /\phi_{w}\right)}^{-\alpha }-s\eta_{r}\left(\phi \right){\tilde{\eta }}_{s}\left(s\right)=0$$

and substituting the solution back to one of Equations (31) or (32) to find $\tilde{r}$. Because $\phi_{w}$ is required to solve the system, with given scalar $\bar{\phi }$, it can be estimated by using the concept of binary search or the so-called “interval halving method”. Since $\phi_{w}$ is always less than the $\bar{\phi }$, we know that the true $\phi_{w}$ is greater than 0 and less than $\bar{\phi }$. By using this information, we can find the the corresponding $\phi_{w}$ that is shown as an intermediate step in Figure 2.

## 3. Results and Discussion

### 3.1. Verification and Validation of Numerical Results

**Figure 3:**
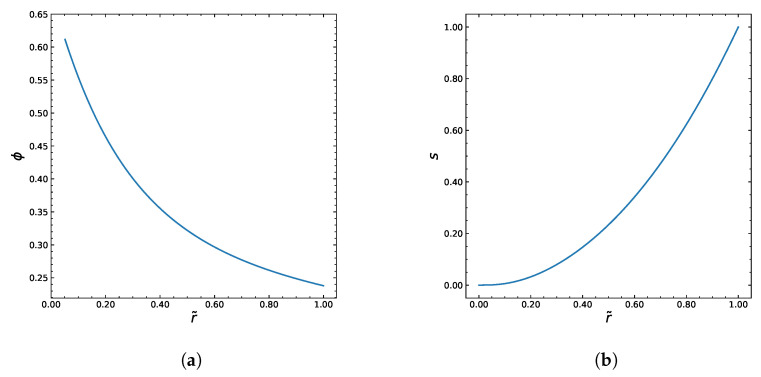
Numerical solutions for microstructural fields obtained from the Carreau–Yasuda-based model. (**a**) Distribution of particle volume fraction $\phi (\tilde{r})$ along the axis of dimensionless radius $\tilde{r}$. (**b**) Graph of the non-dimensional variable $s(\tilde{r})$ v.s. dimensionless radius $\tilde{r}$.

**Figure 4:**
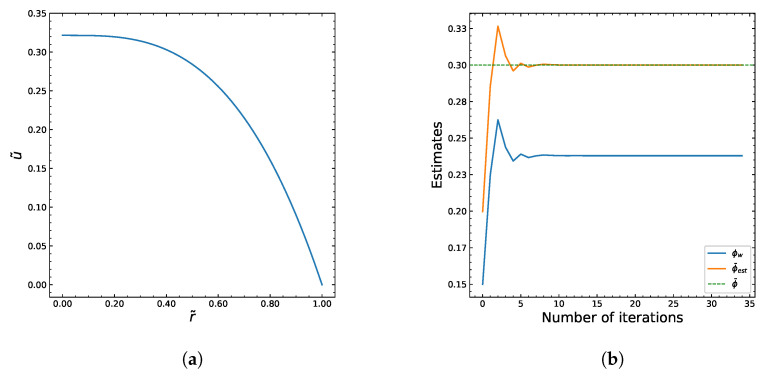
Macroscopic flow characteristics obtained from the numerical algorithm. (**a**) Dimensionless velocity profile $\tilde{u}\left(\tilde{r}\right)$ across the tube radius. (**b**) Convergence history of the iterative scheme for determining the wall particle fraction $\phi_{w}$.

The Carreau–Yasuda rheology enhances particle migration toward the tube centerline, producing stronger core accumulation than in Newtonian suspensions. We aimed to generate some output by using the algorithm shown in Figure 2. The Python programming language was used to implement the numerical algorithm. We assume that the fluid viscosity is described by the Carreau–Yasuda model. The following table represents the constant values we chose to use as a base in the computations. Figure 3 and Figure 4 show the numerical solutions of the system.

**Remark** **1.** *The Carreau–Yasuda parameters $\eta_{0}$, $\eta_{\infty }$, λ, a, and n in Table 1 are adopted from the representative example used by Wang et al. [14], where the reference values $\eta_{0}=1400\;\mathrm{Pa}\cdot s$, λ=1.60s, and a=1.25 are taken directly from their study; in Wang et al., $\eta_{\infty }=0$, but a nonzero value introduces no analytical or numerical difficulty, so we adopt $\eta_{\infty }=100\;\mathrm{Pa}\cdot s$ as a physically plausible lower-shear viscosity plateau. The maximum packing fraction $\phi_{m}=0.68$ represents a typical limit for concentrated suspensions. The ratio $\alpha =K_{c}/K_{\eta }$ is set to 0.66 following the original migration-coefficient values proposed by Phillips et al. [6]. The remaining parameters, including the imposed pressure gradient and radius, are chosen to provide a physically reasonable flow regime for illustrating the numerical behavior of the coupled model in Figure 3 and Figure 4*.

The solutions of particle volume fraction (ϕ) and the dimensionless shear rate (*s*) distributions are shown in Figure 3a,b. The dimensionless velocity ($\tilde{u}$) profile in Figure 4a is computed from the integration of the simple ODE (33). The iterative behavior of the inner algorithm that finds the best approximation of $\phi_{w}$ is shown in Figure 4b.

To verify the numerical model and the corresponding Python code used to solve for ϕ and *s*, we back-substituted these solutions into the system (Equations (31) and (32), labeled as $f_{1}$ and $f_{2}$) and confirmed that the residual errors are on the order of $10^{-16}$. This provides confidence in the numerical accuracy.

Unless otherwise specified, we focus on numerical accuracy and parametric trends, while physical interpretations follow the framework outlined at the beginning of this section.

#### 3.1.1. Newtonian Fluid

**Figure 5:**
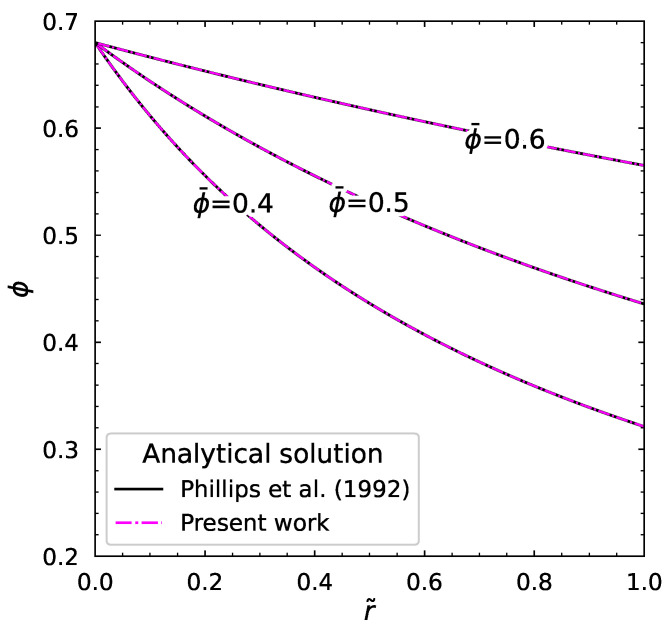
The analytical and numerical solutions of particle volume fraction distribution for different $\bar{\phi }$ [6].

In this section, the validation of our numerical algorithm in comparison with Phillips et al.’s [6] analytical solution is presented. The authors of the analytical solution of the equation governing ϕ in the steady state presented the explicit formula for $K_{c}/K_{\eta }=0.66$ as

(35) $$\phi \left(\tilde{r}\right)=\frac{\phi_{m}}{1+i\tilde{r}}$$

where $i=\frac{\phi_{m}-\phi_{w}}{\phi_{w}}$. This explicit formula describes the particle volume fraction distribution in the steady-state Newtonian case. We compared our numerical algorithm with the analytical solution for different $\bar{\phi }$ and the results are shown in Figure 5.

We can see that our numerical solution perfectly agrees with their results.

#### 3.1.2. Power-Law Fluid

Next, let us consider the viscosity defined by the power-law model as follows

(36) $$\eta \left(\dot{\gamma },\phi \right)={\left(1-\frac{\phi }{\phi_{m}}\right)}^{-1.82}K{\dot{\gamma }}^{n-1}$$

where *K* is the fitted parameter and *n* is the power-law index. We incorporate this equation into our numerical algorithm to solve for concentration profiles for different values of power-law index *n*. To validate our numerical approach, let us derive the analytical solution in a similar way presented by Phillips et al. [6]. From Equation (20) we have

(37) $$\dot{\gamma }={\left[\frac{dp}{dz}\frac{r}{2K\eta_{r}\left(\phi \right)}\right]}^{1/n}$$

and substituting this formula into Equation (17) gives us

(38) $$\frac{\phi }{\phi_{w}}\dot{\gamma }=\frac{{\dot{\gamma_{w}}}^{1+\left(n-1\right)K_{\eta }/K_{c}}}{{\dot{\gamma }}^{\left(n-1\right)K_{\eta }/K_{c}}}{\left[\frac{\eta_{r}\left(\phi_{w}\right)}{\eta_{r}\left(\phi \right)}\right]}^{K_{\eta }/K_{c}}$$

(39) $$\frac{\phi }{\phi_{w}}{\dot{\gamma }}^{1+\left(n-1\right)K_{\eta }/K_{c}}={\dot{\gamma_{w}}}^{1+\left(n-1\right)K_{\eta }/K_{c}}{\left[\frac{\eta_{r}\left(\phi_{w}\right)}{\eta_{r}\left(\phi \right)}\right]}^{K_{\eta }/K_{c}}$$

(40) $$\frac{\phi }{\phi_{w}}\frac{{\dot{\gamma }}^{1+\left(n-1\right)K_{\eta }/K_{c}}}{{\dot{\gamma_{w}}}^{1+\left(n-1\right)K_{\eta }/K_{c}}}={\left[\frac{\eta_{r}\left(\phi_{w}\right)}{\eta_{r}\left(\phi \right)}\right]}^{K_{\eta }/K_{c}}$$

(41) $${\left[\frac{\eta_{r}\left(\phi_{w}\right)}{\eta_{r}\left(\phi \right)}\right]}^{\frac{1}{n}\left(1-K_{\eta }/K_{c}\right)}=\frac{\phi_{w}}{\phi }\frac{1}{{\tilde{r}}^{\frac{1}{n}+\frac{n-1}{n}K_{\eta }/K_{c}}}$$

(42) $${\left[\frac{\phi_{m}-\phi_{w}}{\phi_{m}-\phi }\right]}^{\frac{1}{n}\left(-1.82\right)\left(1-K_{\eta }/K_{c}\right)}=\frac{\phi_{w}}{\phi }\frac{1}{{\tilde{r}}^{\frac{1}{n}+\frac{n-1}{n}K_{\eta }/K_{c}}}$$

By imposing the relation

$$-1.82\left(1-\frac{K_{\eta }}{K_{c}}\right)=1\;\mathrm{and}\;\mathrm{using}\;\mathrm{the}\;\mathrm{empirical}\;\mathrm{ratio}\;\frac{K_{\eta }}{K_{c}}\approx 1.549,$$

we obtain the following analytical expression for the fully developed, steady-state power-law fluid:

(43) $$\phi =\frac{\phi_{w}}{{\tilde{r}}^{\frac{1}{n}\left(1.549n-0.549\right)}}{\left(\frac{\phi_{m}-\phi }{\phi_{m}-\phi_{w}}\right)}^{1/n}$$

The choice in Equation (43) is a specific mathematical condition used to simplify the steady-state shear-induced migration equation and obtain an explicit analytical expression for the particle volume fraction in a power-law fluid. Physically, this corresponds to a situation where the migration flux due to gradients in viscosity ($N_{\eta }$) and the collision-induced flux ($N_{c}$) are related by this particular ratio. While such a precise ratio may not exactly correspond to a specific experimental system, it provides a convenient benchmark for comparison with numerical results and illustrates the influence of the Carreau–Yasuda rheology and particle interactions on the steady-state profile.

**Figure 6:**
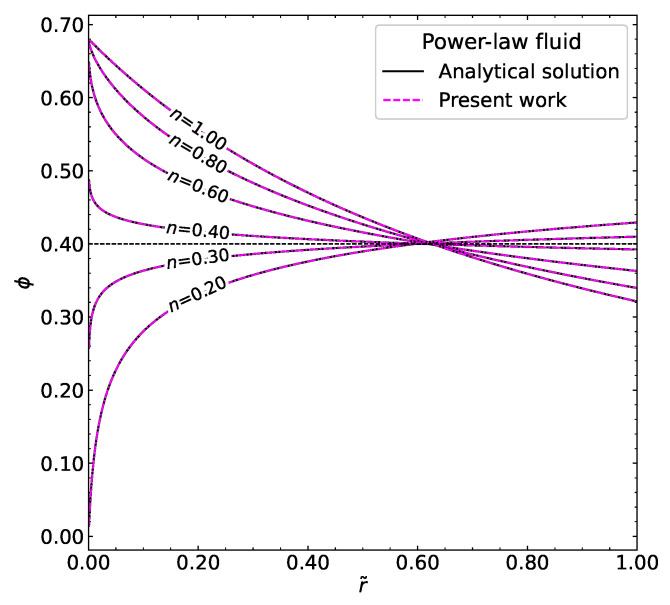
The analytical and numerical solutions of particle volume fraction distribution for different power-law indices *n*.

Figure 6 shows both the analytical solution from the above derivation and our numerical solution for different power-law indices *n*. Interestingly, when (1.549n−0.549)=0, that is n≈0.3544, $\tilde{r}$ vanishes, and the solution of Equation (43) is constant for any value of $\tilde{r}$. For values n⪆0.3544 the concentration profiles are higher near the centerline and lower near the wall of the circular tube; however, if n⪅0.3544 we observe a very small particle volume fraction near the centerline, and it increases towards the wall. From this special case, we can conclude that for $K_{\eta }/K_{c}\approx 1.549$ and n≈0.3544, the particle volume fraction distribution is exactly flat at ϕ=0.40. Generally speaking, when $(1+\left(n-1\right)K_{\eta }/K_{c})=0$ in Equation (43), the concentration profile is $\phi =\bar{\phi }$ along the radial position.

#### 3.1.3. Comparison with Experimental Data

**Figure 7:**
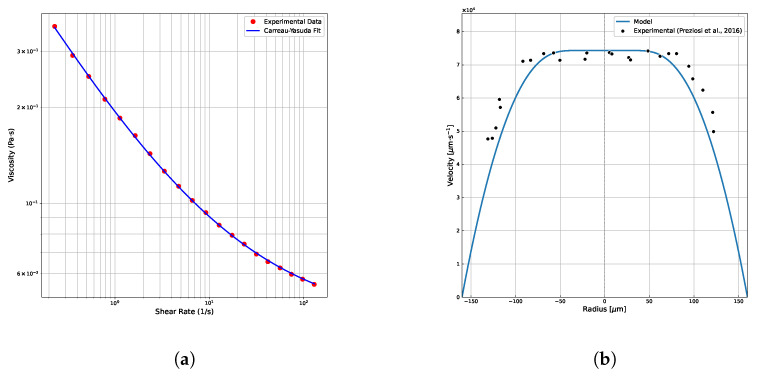
Experimental validation of the numerical model. (**a**) Carreau–Yasuda model fit to experimental viscosity data from [19]. (**b**) Comparison of velocity profiles: experimental data (dots) vs. model prediction (solid line).

To validate the predictive capability of our numerical model, we compared the simulated velocity profile against experimental measurements reported by Preziosi et al. [19] for a low-viscosity emulsion flowing in a confined capillary. In their work, emulsions consisting of silicone oil droplets dispersed in a Boger fluid (high elasticity, HE) were studied under confined Poiseuille flow. Velocity profiles were obtained via high-speed microscopy and droplet tracking. We fitted the Carreau–Yasuda model parameters to the rheological data of the HE fluid provided by Preziosi et al. [19] (see Figure 7a) and used the following parameters in our simulation:

- $\eta_{0}=0.5167$ Pa·s (zero-shear viscosity);
- $\eta_{\infty }=0.0421$ Pa·s (infinite-shear viscosity);
- a=5.0 (Carreau–Yasuda transition parameter);
- λ=10.0 s (relaxation time);
- n=0.5053 (power-law index);
- $\frac{dp}{dz}=1.46\times 10^{6}$ Pa/m (pressure gradient);
- R=0.00016 m (tube radius);
- $\phi_{m}=0.76$ (maximum particle volume fraction used by Preziosi et al.);
- α=0.95 (migration parameter $K_{c}/K_{\eta }$);
- $\bar{\phi }=0.33$ (average particle volume fraction).

Figure 7b shows the comparison between the experimentally measured velocity profile and the profile predicted by our model. The simulation reproduces the experimental trend with good agreement, particularly in the central region of the capillary where the velocity profile exhibits a blunted, plug-like shape due to droplet accumulation. The mean relative error between the predicted and experimental velocities is 7.78%, indicating that the Carreau–Yasuda model combined with the shear-induced migration framework captures the essential rheological and migration behavior of the emulsion. Discrepancies near the wall may be attributed to experimental uncertainties, the simplified assumption of a homogeneous suspension, and possible entry effects not accounted for in our fully developed flow model. Nonetheless, the overall consistency supports the use of our approach for predicting confined suspension flows with elastic matrices.

#### 3.1.4. Other Relative Viscosity Models

**Figure 8:**
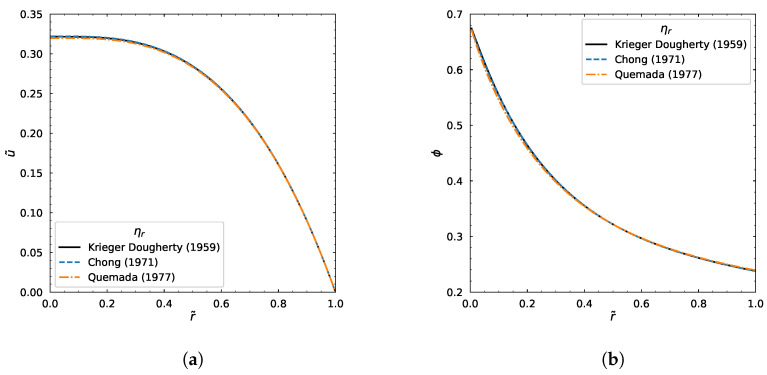
The velocity and concentration profiles using various relative viscosity models [20,21,22]. (**a**) The velocity profiles for various $\eta_{r}$ models. (**b**) The concentration profiles for various $\eta_{r}$ models.

Now let us test our numerical algorithm for various relative viscosity models other than Krieger–Dougherty [20]. The models of Chong (1971) [21] and Quemada (1977) [22] were compared and demonstrated the results of velocity and concentration profiles as seen in Figure 8.

### 3.2. Influence of Average Particle Volume Fraction

Now we analyze the influence of the parameter change on the output. In particular, we will alter one parameter at a time while keeping everything else fixed (as in Table 1) and observe the change in the output. The parameters that will be altered are the average particle volume fraction ($\bar{\phi }$), pressure drop ($\frac{dp}{dz}$), the Carreau–Yasuda model parameters, and the $K_{c}/K_{\eta }$. First, let us consider the effect of average volume fraction on the output results.

**Figure 9:**
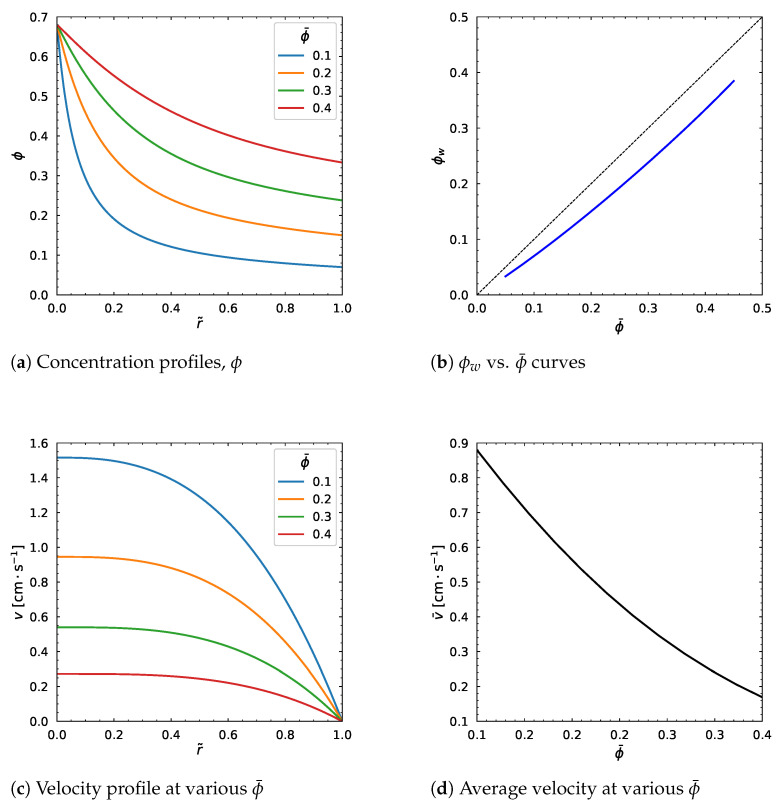
Influence of $\bar{\phi }$. Input values used for the simulation: *R* = 0.01 m, $\phi_{m}$ = 0.68, α = 0.66, $\eta_{\infty }$ = 100 Pa·s, $\eta_{0}$ = 1400 Pa·s, λ = 1.6 s, *a* = 1.25, *n* = 0.5, $\frac{dp}{dz}$ = 0.6 MPa/m.

We change the values of the average particle volume fraction from 0.1 to 0.4 while keeping other parameters fixed (as in Table 1). In Figure 9a, the concentration profile moves upward as $\bar{\phi }$ increases. This observation is as expected given the result in Figure 9b where the relation between $\phi_{w}$ and $\bar{\phi }$ lies below the diagonal line, which agrees with the theory that $0<\phi_{w}<\bar{\phi }$. We can also see that the velocity profile decreases with the increasing average particle volume fraction $\bar{\phi }$ (Figure 9c). Correspondingly, the average velocity decreases as well (Figure 9d). In general, the plots in Figure 9 suggest that the particle volume fraction and velocity distribution are highly dependent on the average particle volume fraction.

### 3.3. Influence of Pressure Gradient

Next, we wanted to determine the influence of pressure drop on the output, so we varied it from $0.2\times 10^{6}$ Pa/m to $1.4\times 10^{6}$ Pa/m and kept everything else constant (as in Table 1).

**Figure 10:**
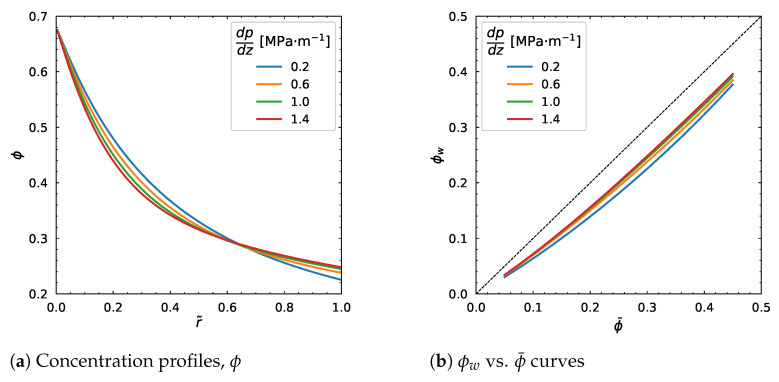
Effect of pressure gradient, $\frac{dp}{dz}$. Input values used for the simulation: *R* = 0.01 m, $\phi_{m}$ = 0.68, α = 0.66, $\eta_{\infty }$ = 100 Pa·s, $\eta_{0}$ = 1400 Pa·s, λ = 1.6 s, *a* = 1.25, *n* = 0.5, $\bar{\phi }$ = 0.30.

**Figure 11:**
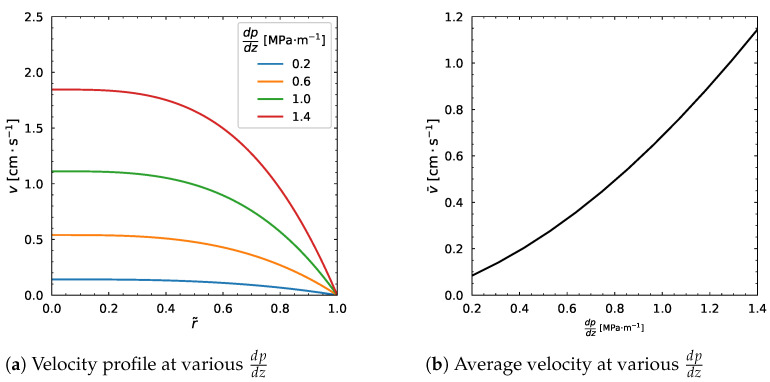
Influence of pressure gradient, $\frac{dp}{dz}$. Input values used for the simulation: *R* = 0.01 m, $\phi_{m}$ = 0.68, α = 0.66, $\eta_{\infty }$ = 100 Pa·s, $\eta_{0}$ = 1400 Pa·s, λ = 1.6 s, *a* = 1.25, *n* = 0.5, $\bar{\phi }$ = 0.30.

The behavior of the concentration profile is not uniform since, in Figure 10a, it decreases near the centerline ($\tilde{r}=0$) but increases near the wall ($\tilde{r}=1$) as the pressure drop increases. Clearly, the $\phi_{w}$ vs. $\bar{\phi }$ curve moves upwards as shown in Figure 10b, and the velocity profile increases as well as the average velocity (Figure 11a,b) with the increasing pressure drop. Overall, we can see that seemingly the pressure drop affects the velocity profile but has weak relation to the particle volume fraction.

### 3.4. Influence of Carreau–Yasuda Parameters

Our next analysis examines the effect of the viscosity at infinite shear rate, $\eta_{\infty }$, which was varied from 100 to 700 Pa·s.

**Figure 12:**
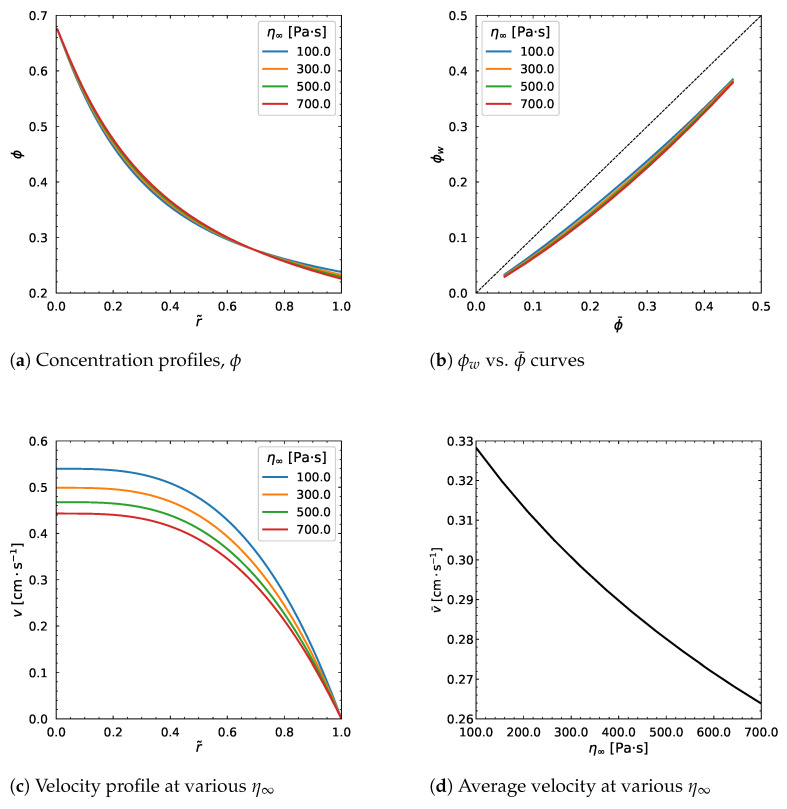
Influence of $\eta_{\infty }$. Input values used for the simulation: *R* = 0.01 m, $\phi_{m}$ = 0.68, α = 0.66, $\eta_{0}$ = 1400 Pa·s, λ = 1.6 s, *a* = 1.25, *n* = 0.5, $\frac{dp}{dz}$ = 0.6 MPa/m, $\bar{\phi }$ = 0.30.

We can notice that the higher value of $\eta_{\infty }$ leads to a slight increase near the center and slight decrease in particle volume fraction distribution (ϕ) near the wall in Figure 12a. The $\phi_{w}$ and $\bar{\phi }$ relation curve moves downwards as shown in Figure 12b. The velocity decreases gradually as well as the average velocity with the increase in $\eta_{\infty }$ (Figure 12c,d).

**Figure 13:**
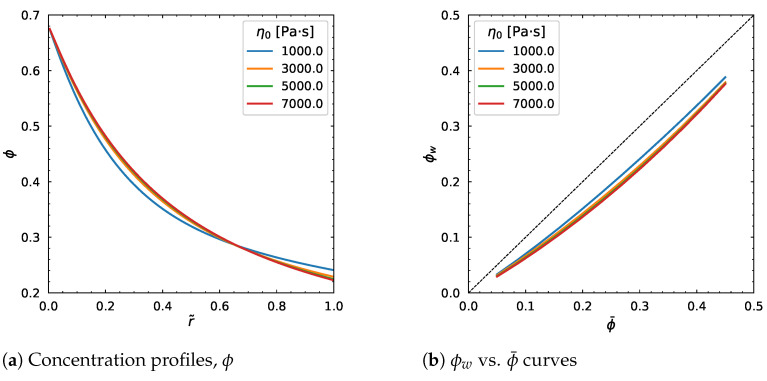

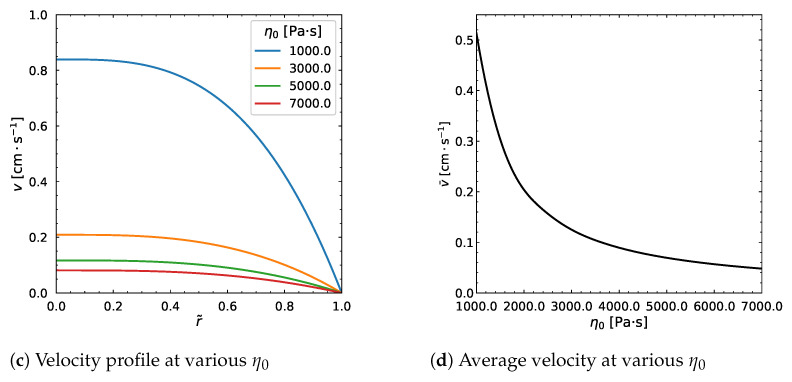
Effect of $\eta_{0}$. Input values used for the simulation: *R* = 0.01 m, $\phi_{m}$ = 0.68, α = 0.66, $\eta_{\infty }$ = 100 Pa·s, λ = 1.6 s, *a* = 1.25, *n* = 0.5, $\frac{dp}{dz}$ = 0.6 MPa/m, $\bar{\phi }$ = 0.30.

**Figure 14:**
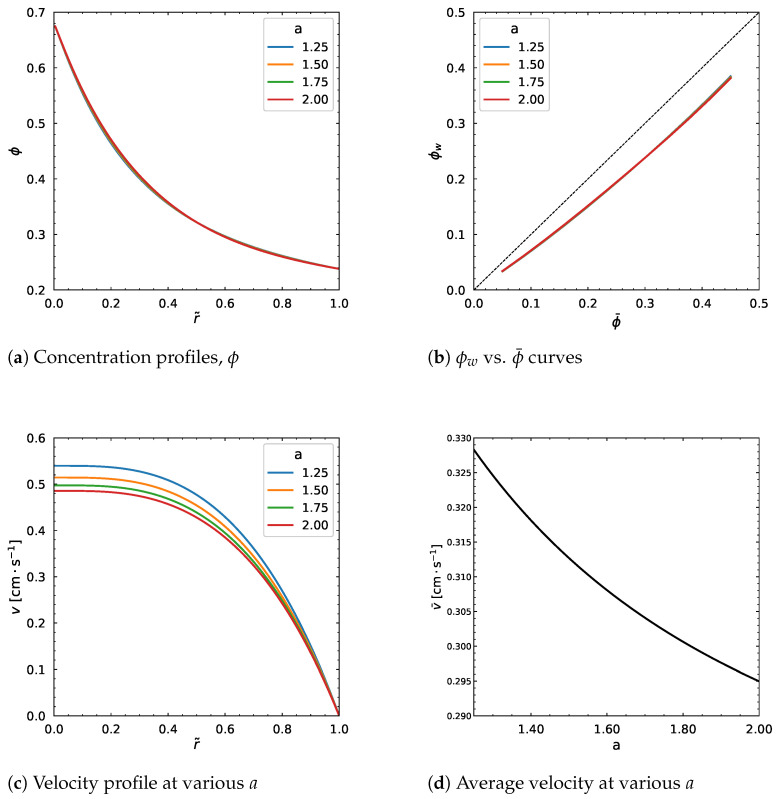
Effect of *a*. Input values used for the simulation: *R* = 0.01 m, $\phi_{m}$ = 0.68, α = 0.66, $\eta_{\infty }$ = 100 Pa·s, $\eta_{0}$ = 1400 Pa·s, λ = 1.6 s, *n* = 0.5, $\frac{dp}{dz}$ = 0.6 MPa/m, $\bar{\phi }$ = 0.30.

A strong impact is observed on the velocity profile, whereas the concentration profile is nearly unaffected. The same behavior is shown in Figure 13 when we increase the $\eta_{0}$ from 1000 Pa·s to 7000 Pa·s. No considerable influence results from the change in the dimensionless parameter *a* as can be seen from Figure 14. In particular, the velocity profile decreases by increasing *a*, but stops changing regardless of the change in the parameter (Figure 14c), which is same for the average velocity.

**Figure 15:**
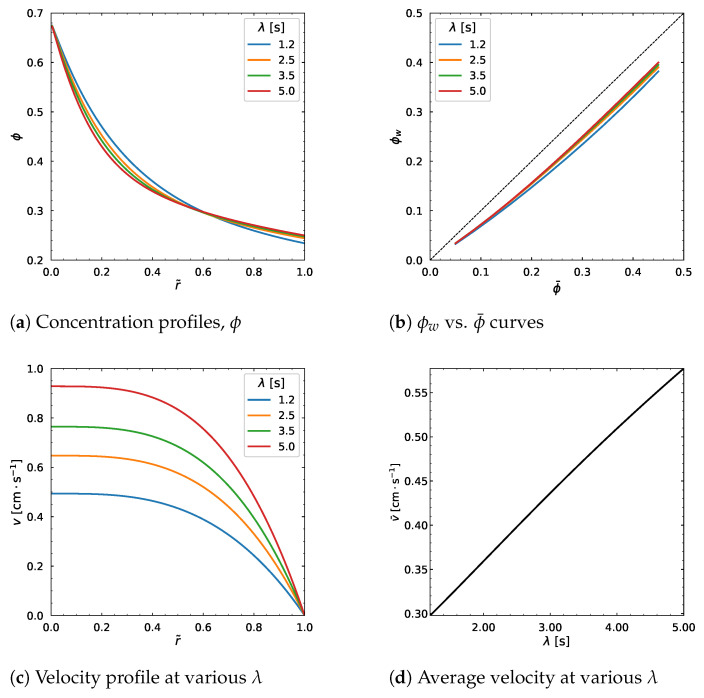
Effect of λ. Input values used for the simulation: *R* = 0.01 m, $\phi_{m}$ = 0.68, α = 0.66, $\eta_{\infty }$ = 100 Pa·s, $\eta_{0}$ = 1400 Pa·s, *a* = 1.25, *n* = 0.5, $\frac{dp}{dz}$ = 0.6 MPa/m, $\bar{\phi }$ = 0.30.

Interestingly, the time constant λ of the Carreau–Yasuda model affects the output of ϕ and *v* curves to a high extent. When we vary the λ parameter from 1.2 s to 5 s, in Figure 15a, the ϕ distribution decreases near the centerline ($\tilde{r}=0$) and increases near the wall ($\tilde{r}=1$). However almost no change is observed for $\tilde{r}=0.6$.

The relation curve between $\phi_{w}$ and $\bar{\phi }$ moves upward as shown in Figure 15 b, and the velocity profile as well as the average velocity linearly increase with the increasing λ as demonstrated in Figure 13d and Figure 15c.

**Figure 16:**
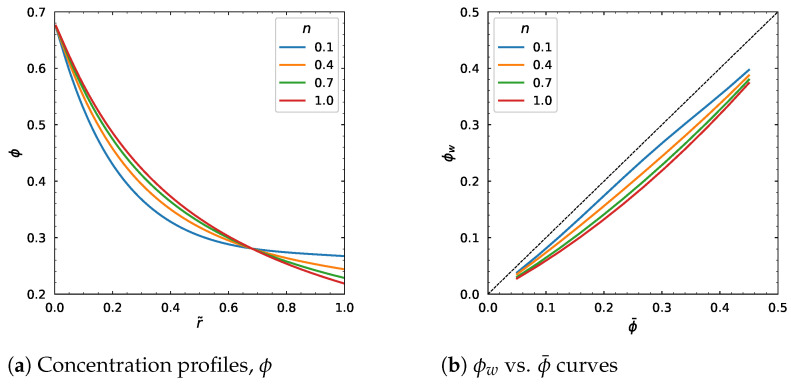

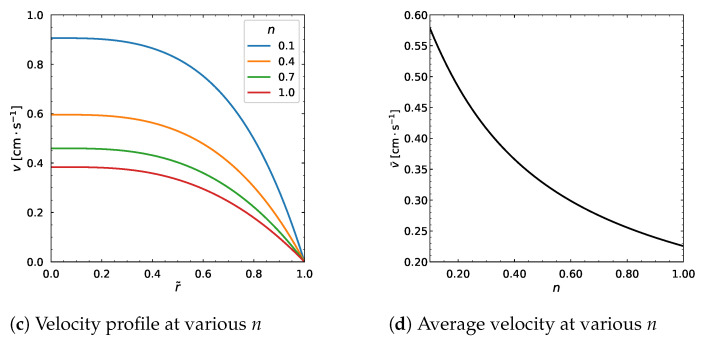
Effect of *n*. Input values used for the simulation: *R* = 0.01 m, $\phi_{m}$ = 0.68, α = 0.66, $\eta_{\infty }$ = 100 Pa·s, $\eta_{0}$ = 1400 Pa·s, λ = 1.6 s, *a* = 1.25, $\frac{dp}{dz}$ = 0.6 MPa/m, $\bar{\phi }$ = 0.30.

The parameter *n* was varied from 0.1 to 1, and the concentration profile is lower near the wall and is higher near the center as shown in Figure 16a. This means that if we assume the Newtonian fluid rheology, this would model the concentration distribution with higher values closer to the center and lower values closer to the wall. The velocity profile and average velocity decrease as *n* approaches 1 (Figure 16c,d).

### 3.5. Influence of Other Model Parameters

**Figure 17:**
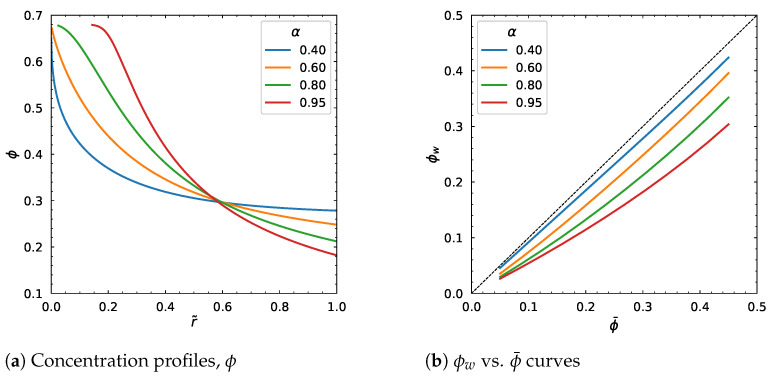
Effect of α parameter. Input values used for the simulation: *R* = 0.01 m, $\phi_{m}$ = 0.68, $\eta_{\infty }$ = 100 Pa·s, $\eta_{0}$ = 1400 Pa·s, λ = 1.6 s, *a* = 1.25, *n* = 0.5, $\frac{dp}{dz}$ = 0.6 MPa/m, $\bar{\phi }$ = 0.30.

**Figure 18:**
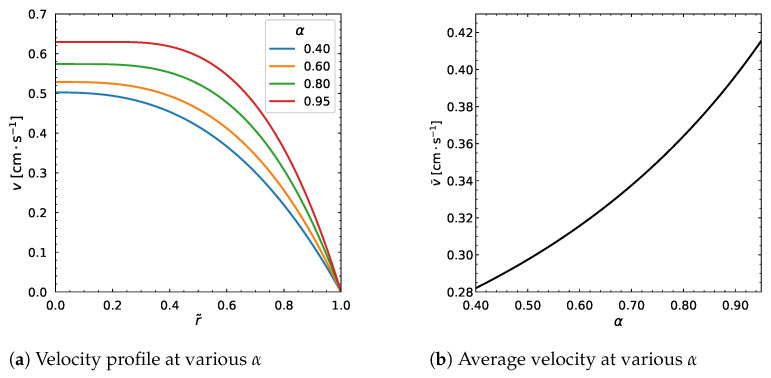
Effect of α parameter. Input values used for the simulation: *R* = 0.01 m, $\phi_{m}$ = 0.68, $\eta_{\infty }$ = 100 Pa·s, $\eta_{0}$ = 1400 Pa·s, λ = 1.6 s, *a* = 1.25, *n* = 0.5, $\frac{dp}{dz}$ = 0.6 MPa/m, $\bar{\phi }$ = 0.30.

The fitting parameter, $\alpha =K_{c}/K_{\eta }$, took the values from 0.4 to 0.95. This change affected the output considerably (Figure 17 and Figure 18). In particular, the particle volume fraction distribution is higher near the center and is lower near the wall. There is a point at about $\tilde{r}=0.6$ where ϕ does not change. The velocity profile and average velocity increase gradually as α increases.

The behavior observed in Figure 16a for small $\tilde{r}$ and large α (red curve) is primarily numerical rather than physical. Equations (31) and (32) and the single-equation form (34) show that for high α, the particle concentration ϕ can become very large near the y-axis (center of the tube), approaching the maximum packing fraction $\phi_{m}$.

Through constitutive relations such as Krieger–Dougherty, this leads to a rapid increase in the effective viscosity η(ϕ), and the governing relation for the shear rate becomes extremely stiff. In addition, the cylindrical-coordinate terms (effectively containing $1/\tilde{r}$) require careful regularization at $\tilde{r}\to 0$.

Numerically, the combination of (1) ϕ approaching $\phi_{m}$ (causing large viscosity), (2) steep gradients amplified by large α, and (3) singular $1/\tilde{r}$, can make the behavior at the axis to produce overflow/NaN or cause the integrator to reject steps near the axis, which appears as missing points for the red curve in the plotted results in Figure 17.

## 4. Conclusions

In this study, fully developed fluid flows with filled particles in a circular tube were analyzed. The Carreau–Yasuda and Krieger & Dougherty models were used to describe the particle-filled fluid and effective viscosities. respectively. The well-known diffusive flux model, proposed by Phillips et al. [6], was taken as a base of our analysis. The model was simplified by means of the non-dimensionalization technique, and a corresponding numerical method was applied for the solution. The verification was achieved by back-substituting the solution to the model that showed very small error. With the simplicity of our approach, the influence of particle and flow distributions by the model and the rheological parameters were observed. In particular, the particle concentration and velocity profiles depend strongly on λ and *n* (Carreau–Yasuda parameters), the migration parameter $\alpha =K_{c}/K_{\eta }$, and the average volume fraction $\bar{\phi }$. The particle volume fraction distribution was weakly varied by the change in pressure gradient $\frac{dp}{dz}$ and rheology parameters $\eta_{\infty }$, $\eta_{0}$ and *a*, but they strongly affected the velocity profile.

Future research can apply this method for determining particle volume fractions in other channels such as rectangular or curved channels. The limitation of our study is that our method is applied to only circular domains and can be extended to non-circular channels. This method could be particularly useful for studying similar fluid dynamics in more complex scenarios, such as coating and extrusion die manifolds, where the behavior of particle-filled fluids in confined geometries presents additional challenges and opportunities for analysis.

## Figures and Tables

**Table 1:** Constant parameters used in the Carreau–Yasuda model and flow simulations.

Parameters	$\eta_{0}$ (Pa·s)	$\eta_{\infty }$ (Pa·s)	*a*	λ (s)	*n*	$\frac{\mathrm{dp}}{\mathrm{dz}}$ (MPa/m)	*R* (m)	$\phi_{m}$	$\alpha =K_{c}/K_{\eta }$	$\bar{\phi }$
	1400	100	1.25	1.6	0.5	0.6	0.01	0.68	0.66	0.3

**Figure A1:**
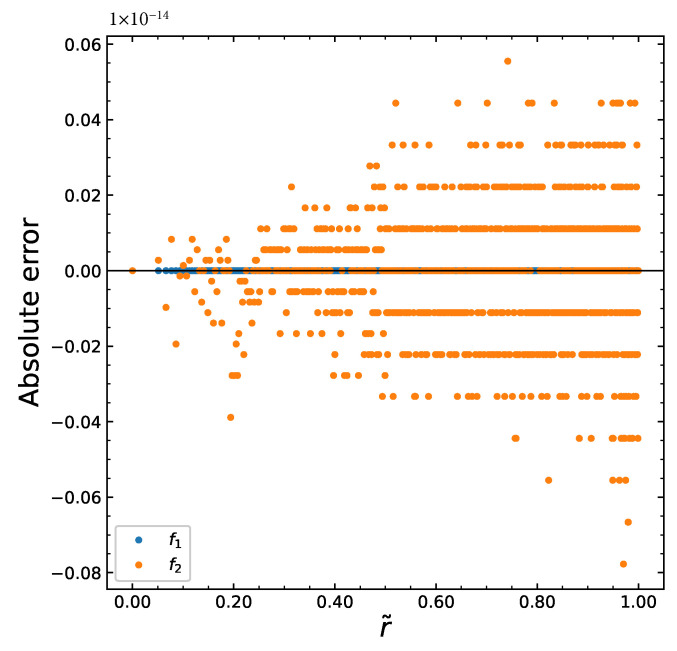
Absolute errors of the system with the Carreau–Yasuda viscosity model as a function of the non-dimensional radius $\tilde{r}$. The vertical dispersion of points at a given $\tilde{r}$ arises because multiple values are reported across grid points. The magnitude of these errors, on the order of $10^{-14}$, is close to machine precision, so the scatter does not indicate a physical effect but rather numerical round-off and discretization tolerance.

**Figure A2:**
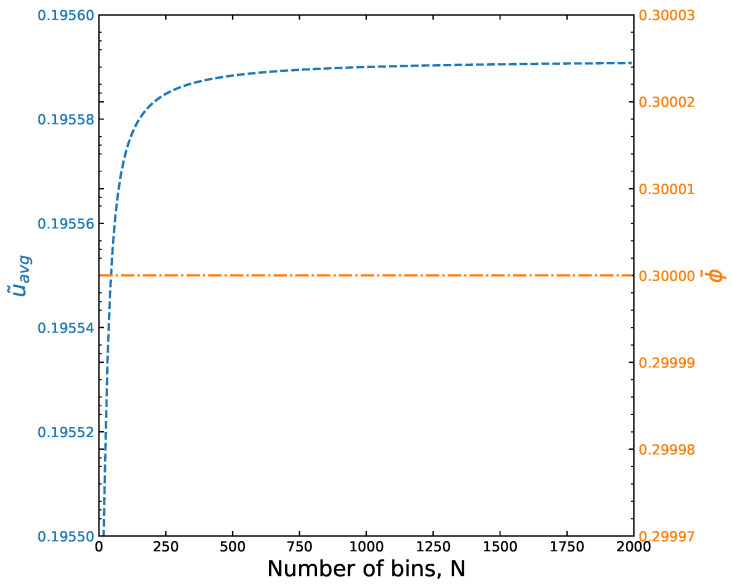
Average velocity and average particle volume fraction at various numbers of bins.

**Figure A3:**
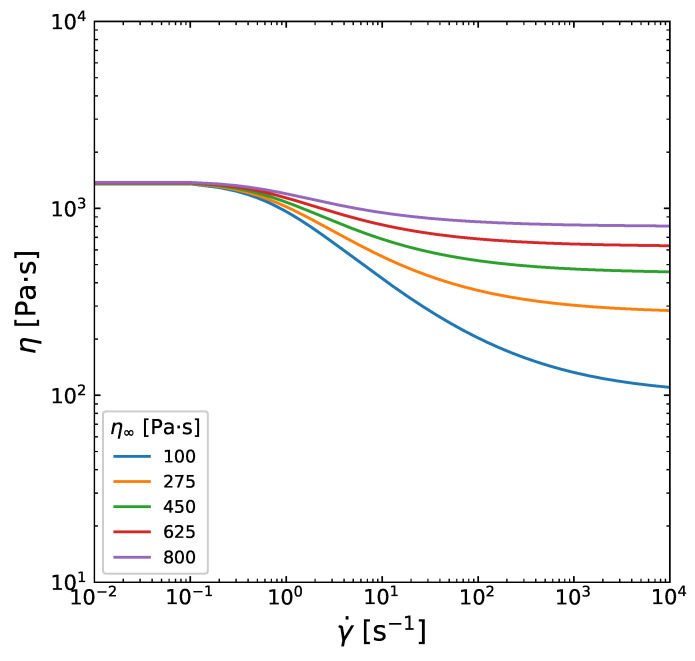
Influence of change in $\eta_{\infty }$ on the viscosity.

**Figure A4:**
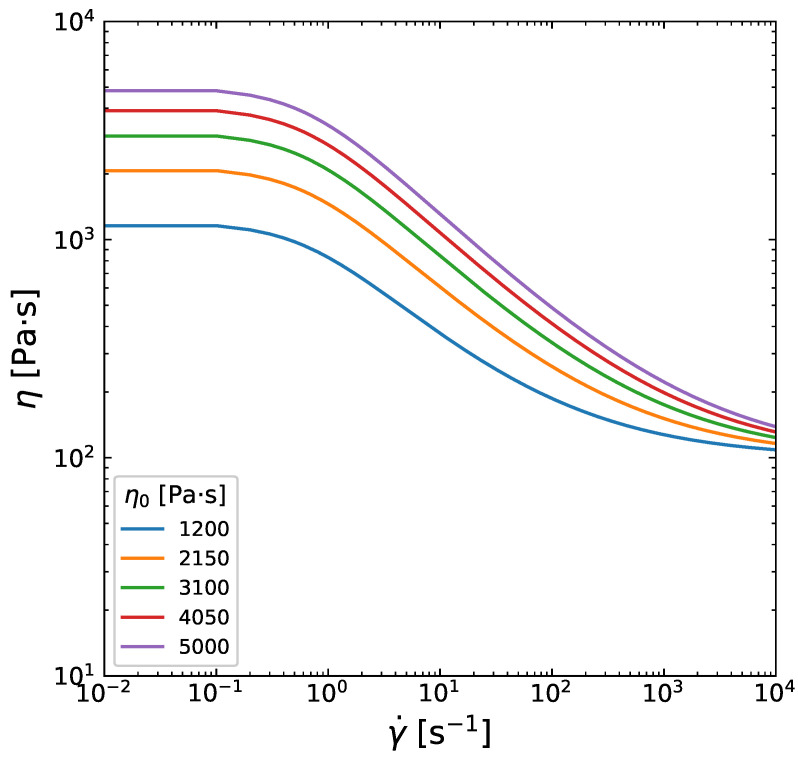
Influence of change in $\eta_{0}$ on the viscosity.

**Figure A5:**
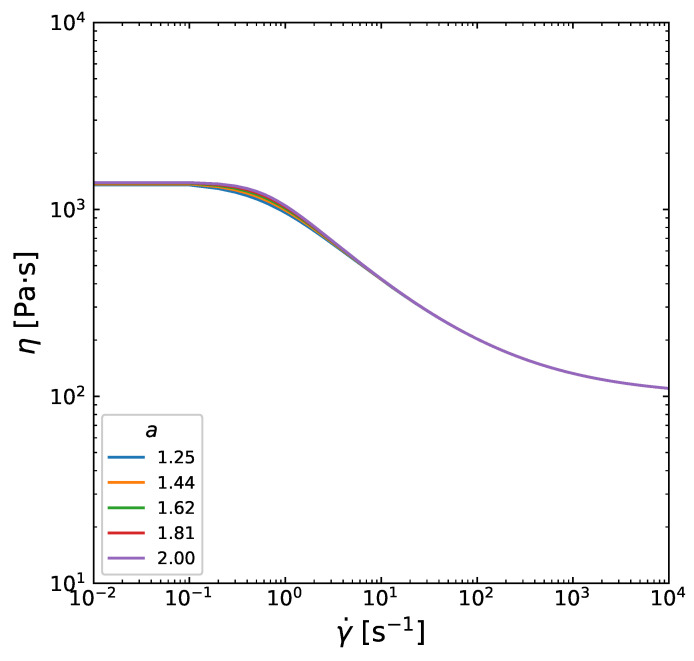
Influence of change in *a* on the viscosity.

**Figure A6:**
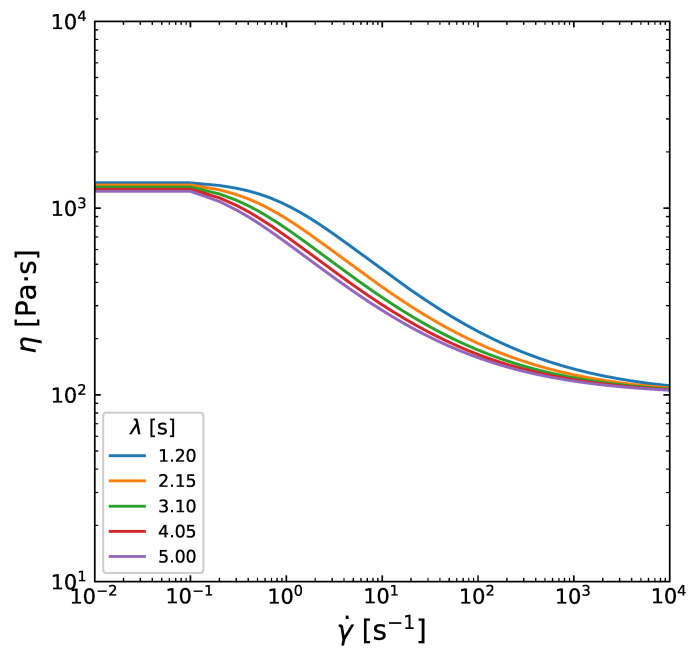
Influence of change in λ on the viscosity.

**Figure A7:**
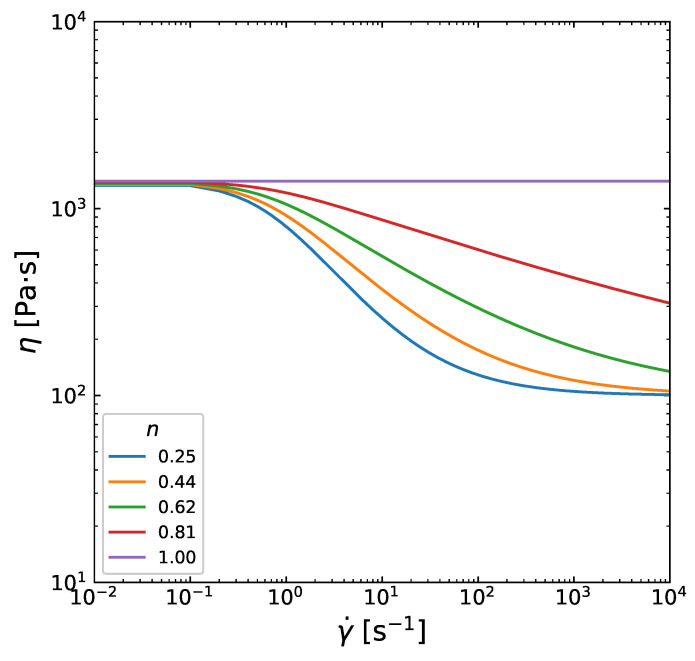
Influence of change in *n* on the viscosity.

## Data Availability

The data presented in the study are openly available in [GitHub] at [https://github.com/medeuamangeldi/particle-laden-flows].

## Nomenclature

ϕ	Particle volume fraction
$\phi_{m}$	Maximum packing fraction
$\bar{\phi }$	Average particle volume fraction
$\phi_{w}$	Particle volume fraction at the wall
*r*	Point along radius of the tube (m)

$\tilde{r}$	Point along nondimensional radius of the tube
*R*	Maximum radius of the tube (m)
*z*	Axial coordinate (m)
$\dot{\gamma }$	Shear rate ($s^{-1}$)
${\dot{\gamma }}_{w}$	Shear rate at the wall ($s^{-1}$)
*s*	Nondimensional shear rate
$\tilde{u}$	Nondimensional velocity
*v*	Dimensional velocity (cm/s)
$\bar{v}$	Dimensional average velocity (cm/s)
$\frac{dp}{dz}$	Pressure gradient (Pa/m)
η	Viscosity (Pa·s)
$\eta_{w}$	Viscosity at the wall (Pa·s)
$\tilde{\eta }$	Nondimensional viscosity
$\eta_{0}$	Zero-shear viscosity (Pa·s)
$\eta_{\infty }$	Infinite-shear viscosity (Pa·s)
λ	Time constant (s)
*n*	Power-law index (–)
*a*	Yasuda parameter (–)
$K_{c}$	Particle migration coefficient due to collisions
$K_{\eta }$	Particle migration coefficient due to viscosity gradients
∇ϕ	Gradient of particle concentration (1/m)
σ	Shear stress (Pa)
τ	Extra stress tensor (Pa)
*b*	particle radius (m)

## Appendix C. Derivation of the Reduced Steady-State System

Starting from the shear-induced diffusion equation

$$J=-K_{c}a^{2}\phi \dot{\gamma }\nabla \phi -K_{\eta }a^{2}\phi^{2}\dot{\gamma }\nabla \ln\eta ,\;\nabla \cdot J=0,$$

and assuming axisymmetric fully developed flow in a circular tube, the radial flux balance becomes

$$\frac{d}{dr}\left(-K_{c}\phi \dot{\gamma }\frac{d\phi }{dr}-K_{\eta }\phi^{2}\dot{\gamma }\frac{d}{dr}\ln\eta \right)=0.$$

Since the flux must vanish everywhere, setting the term inside the derivative equal to zero yields

$$K_{c}\;\phi^{\prime }\;+K_{\eta }\;\phi \;{\left(\ln\eta \right)}^{\prime }=0,$$

which simplifies to the second equation in (21).

The momentum equation for steady, axisymmetric flow is

$$\frac{1}{r}\frac{d}{dr}\;\left(r\;\eta \dot{\gamma }\right)=-\frac{dp}{dz}.$$

Integrating once gives the first equation in (21),

$$\eta \dot{\gamma }=-\frac{dp}{dz}\frac{r}{2}.$$

Introducing the nondimensional variables in Equation (22) and substituting them into the above relations yields Equations (23)–(28). Finally, the constitutive viscosity laws,

$$\eta \left(\phi ,\dot{\gamma }\right)=\eta_{r}\left(\phi \right)\;\eta_{s}\left(\dot{\gamma }\right),$$

together with Equation (24), close the system.
